# Diagnostic and Prognostic Value of Focused Assessment With Sonography in Trauma (FAST) in Hemorrhage Stratification and Trauma Severity Classification

**DOI:** 10.7759/cureus.96430

**Published:** 2025-11-09

**Authors:** Mohammed Usman Khan Wazir, Kiranjot Kaur, Zainab Shehzadi, Ahmed M Mohamed, Shashwat Shetty, Beshr Mosa Basha, Muhammad Faizan Butt, Shahmeen Rasul, Mustafa Makkiyah, Ayesha Farooqi

**Affiliations:** 1 Trauma and Orthopaedics Surgery, Northwick Park Hospital, London, GBR; 2 Medicine, United States Navy, United States Military, North Chicago, USA; 3 Clinical Research, Arizona State University, Tempa, USA; 4 Internal Medicine, Shri B.M. Patil Medical College, Bijapur, IND; 5 Medicine, Sharif Medical and Dental College, Lahore, PAK; 6 Trauma, University of Gezira, Wad Madani, SDN; 7 Orthopaedics, The Hillingdon Hospitals NHS Foundation Trust, London, GBR; 8 General Surgery, Dr. Sulaiman Al Habib Medical Group, Khobar, SAU; 9 Medical Education, NHS Highland, Inverness, GBR; 10 Trauma and Orthopaedics, University Hospitals of Derby and Burton NHS Foundation Trust, Derby, GBR; 11 Trauma and Orthopaedics, The Hillingdon Hospitals NHS Foundation Trust, London, GBR; 12 General Surgery, Dow University of Health Sciences, Civil Hospital Karachi, Karachi, PAK

**Keywords:** emergency surgery, fast, hemorrhage, prognosis, stratification, trauma, trauma severity, ultrasonography

## Abstract

Uncontrolled hemorrhage is the leading preventable cause of early death after trauma. The Focused Assessment with Sonography in Trauma (FAST) examination is widely used to detect free intraperitoneal fluid, but its prognostic value for hemorrhage grading and trauma severity classification requires synthesis. A systematic review was conducted following the Preferred Reporting Items for Systematic Reviews and Meta-Analyses (PRISMA) 2020 guidelines. PubMed, Embase, Scopus, and Cochrane databases were searched through March 2025 for human studies assessing the diagnostic accuracy and prognostic utility of FAST in blunt or penetrating trauma. Studies were selected based on the population (or patient/problem), intervention, comparison (or control), and outcome (PICO) framework, excluding case reports, editorials, conference abstracts, animal studies, and those without extractable outcomes. Risk of bias was assessed using the Newcastle-Ottawa Scale (NOS) and the Quality Assessment of Diagnostic Accuracy Studies, Version 2 (QUADAS-2) tool. Seven studies, including approximately 7,310 patients, met the inclusion criteria. FAST demonstrated high specificity up to 97% for detecting clinically significant hemoperitoneum. Positive findings were associated with increased transfusion requirements, greater need for surgery or embolization, higher intensive care unit (ICU) admissions, elevated injury severity, and higher short-term mortality. Semi-quantitative interpretations, such as the number and distribution of positive zones, enhanced hemorrhage stratification. Combining FAST with physiological parameters in validated trauma scoring systems improved the prediction of massive transfusion and operative urgency. Despite operator dependence and limited sensitivity for small or retroperitoneal bleeds, standardized protocols and serial assessments can strengthen its role in early trauma decision-making.

## Introduction and background

Trauma continues to be a leading cause of global morbidity and mortality, accounting for over five million deaths annually and representing the most common cause of death in individuals under 45 years of age [1]. Among trauma-related fatalities, uncontrolled hemorrhage is recognized as the most preventable cause of early death, with nearly 40% of trauma-related mortality attributable to bleeding, particularly within the first “golden hour” following injury [2]. Rapid identification and stratification of hemorrhage severity are, therefore, critical in the emergency setting to guide timely fluid resuscitation, transfusion protocols, and operative decision-making. Focused Assessment with Sonography in Trauma (FAST) has become an indispensable diagnostic modality in emergency medicine. It is a portable, non-invasive, and rapid bedside tool designed to detect free fluid in the peritoneal, pericardial, and pleural spaces [3]. While its diagnostic role in identifying hemoperitoneum is well established, growing evidence highlights its prognostic value, as positive FAST findings correlate with increased transfusion requirements, operative intervention, higher injury severity score (ISS), and mortality [4]. Thus, FAST provides not only a diagnostic framework but also contributes to risk stratification and outcome prediction in severely injured patients. Several classification systems exist to guide hemorrhage assessment.

The Advanced Trauma Life Support (ATLS) hemorrhagic shock classification divides blood loss into four classes based on volume, hemodynamics, and clinical features. Class I (<750 mL, <15%) is usually well compensated with minimal signs. Class II (750-1500 mL, 15-30%) presents with tachycardia and narrowed pulse pressure but preserved systolic pressure. Class III (1500-2000 mL, 30-40%) causes hypotension, tachypnea, oliguria, and confusion, while Class IV (>2000 mL, >40%) results in profound hypotension, negligible urine output, and altered consciousness [5]. FAST findings, although not a formal classification system, have been integrated into prognostic models and scoring tools. Semi-quantitative approaches such as the Kirkpatrick FAST scoring system provide a semi-quantitative assessment of intra-abdominal bleeding by assigning prognostic weight to the number of positive sonographic zones, including Morison’s pouch, the pelvis, pericardium, and pleural spaces. Patients with two or more positive zones have a significantly higher likelihood of severe hemorrhage, need for laparotomy, and increased mortality, making this system useful in stratifying operative urgency [6].

Similarly, the Kimura and Boulanger sonographic grading of hemoperitoneum categorizes free fluid into three grades: Grade 1, fluid confined to a single anatomical area; Grade 2, fluid in multiple regions with thin layers; and Grade 3, large-volume collections or complex findings such as clotted blood or floating bowel loops. Higher grades correlate with greater blood loss, increased transfusion requirements, and a higher probability of surgical intervention [7]. Both scoring systems move beyond the binary positive/negative FAST result, providing clinicians with prognostic information that enhances decision-making in hemorrhage stratification and trauma severity classification. Moreover, validated trauma scores such as the Trauma-Associated Severe Hemorrhage (TASH) score and the Assessment of Blood Consumption (ABC) score incorporate FAST results with clinical and laboratory variables to predict massive transfusion requirements [8,9]. In practice, these tools complement each other: ATLS hemorrhage classes provide a physiological context, FAST-based scoring offers anatomical and semi-quantitative assessment, and integrated systems such as TASH and ABC link FAST findings with hemodynamic parameters to predict transfusion and operative needs. Together, they reinforce FAST as both a diagnostic and prognostic tool in trauma care. This review, therefore, aimed to evaluate the diagnostic accuracy and prognostic utility of FAST in hemorrhage stratification and trauma severity classification.

## Review

Materials and methods

Search Strategy

A systematic literature search was performed across four electronic databases: PubMed, Embase, Scopus, and the Cochrane Library, in accordance with the Preferred Reporting Items for Systematic Reviews and Meta-Analyses (PRISMA) 2020 statement to ensure methodological transparency and reproducibility [10]. The search strategy incorporated both controlled vocabulary (e.g., MeSH, Emtree terms) and free-text keywords related to trauma and ultrasonography. Terms included “FAST”, “Focused Assessment with Sonography for Trauma”, “hemorrhage”, “abdominal trauma”, “trauma severity”, and “prognosis”. Boolean operators (AND, OR) were used to optimize the sensitivity and specificity of the search. The search was restricted to human studies and included all publications available up to March 2025. No language restrictions were applied to minimize selection bias. In addition to database queries, the reference lists of all eligible studies and relevant reviews were manually screened to identify additional citations. Abstracts without accessible full texts, duplicate publications, and studies lacking extractable outcome data were excluded. The search strategy was designed to be statistically robust by maximizing sensitivity (capturing all potentially relevant studies) while preserving specificity (excluding irrelevant literature). The number of records retrieved from each database, screened, and excluded was documented using a PRISMA flow diagram, ensuring full reporting of the study selection process [10].

Eligibility Criteria

The eligibility of studies was defined using the population (or patient/problem), intervention, comparison (or control), and outcome (PICO) framework to directly address the diagnostic and prognostic value of FAST in hemorrhage stratification and trauma severity classification [11]. Eligible studies included human patients presenting with blunt or penetrating trauma who underwent assessment for hemorrhage using FAST, performed either in the emergency department or in prehospital settings. Comparisons were made with FAST-negative patients or with gold-standard reference modalities such as computed tomography (CT), laparotomy, or autopsy. The primary outcomes of interest were diagnostic accuracy measures, including sensitivity, specificity, predictive values, and likelihood ratios for the detection of hemorrhage. Secondary outcomes encompassed prognostic indicators such as mortality, transfusion requirements, intensive care unit (ICU) admission, surgical or interventional procedures, and the integration of FAST findings into hemorrhage stratification and trauma severity classification models. Studies were excluded if they were case reports, conference abstracts, editorials, narrative reviews, animal studies, or if they lacked extractable outcome data relevant to either diagnostic accuracy or prognostic endpoints.

Study Selection

Two independent reviewers screened titles and abstracts, followed by full-text assessment of potentially eligible articles. Discrepancies were resolved through consensus or, when necessary, arbitration by a third reviewer. The PRISMA flow diagram was used to document the study selection process, including the number of records identified, screened, excluded, and finally included in the review.

Data Extraction

Two reviewers independently extracted data using a standardized template following PRISMA 2020 guidelines. Information collected included study design, setting, sample size, patient demographics, trauma mechanism, and details of the FAST examination (timing, operator, positivity criteria). Comparator standards (CT, laparotomy, autopsy) and outcome measures were also recorded. Diagnostic accuracy parameters (sensitivity, specificity, predictive values, likelihood ratios) and prognostic outcomes (mortality, transfusion needs, ICU admission, surgical intervention, trauma severity scores) were extracted. Discrepancies were resolved by consensus, and incomplete data were cross-checked with supplementary materials or related publications.

Risk of Bias Assessment

The methodological quality of included studies was appraised using the Newcastle-Ottawa Scale (NOS) for cohort and case-control studies [12] and the Quality Assessment of Diagnostic Accuracy Studies, Version 2 (QUADAS-2) tool for diagnostic accuracy studies [13]. These frameworks assess potential bias in patient selection, index test interpretation, reference standard validity, and study flow. Each study was assigned a risk of bias rating (low, moderate, or high) based on predefined criteria.

Data Synthesis

Data were extracted into standardized forms and synthesized narratively and in tabular format. Extracted variables included study design, sample size, patient population, FAST methodology, comparator standards, and outcome measures. Where available, diagnostic accuracy parameters (sensitivity, specificity, likelihood ratios) were tabulated. Prognostic associations with mortality, transfusion requirements, operative interventions, and trauma severity indices were highlighted. Given the heterogeneity in study design, populations, and reported outcomes, a meta-analysis was not performed, and synthesis was limited to descriptive and comparative analysis.

Registration/Guideline

This review was conducted in accordance with PRISMA 2020 recommendations; however, it was not prospectively registered in the International Prospective Register of Systematic Reviews (PROSPERO) due to its narrative and scoping orientation. Future reviews incorporating meta-analytical methods should consider prospective registration to enhance methodological rigor and transparency.

Results

Study Selection Process

**Figure 1:**
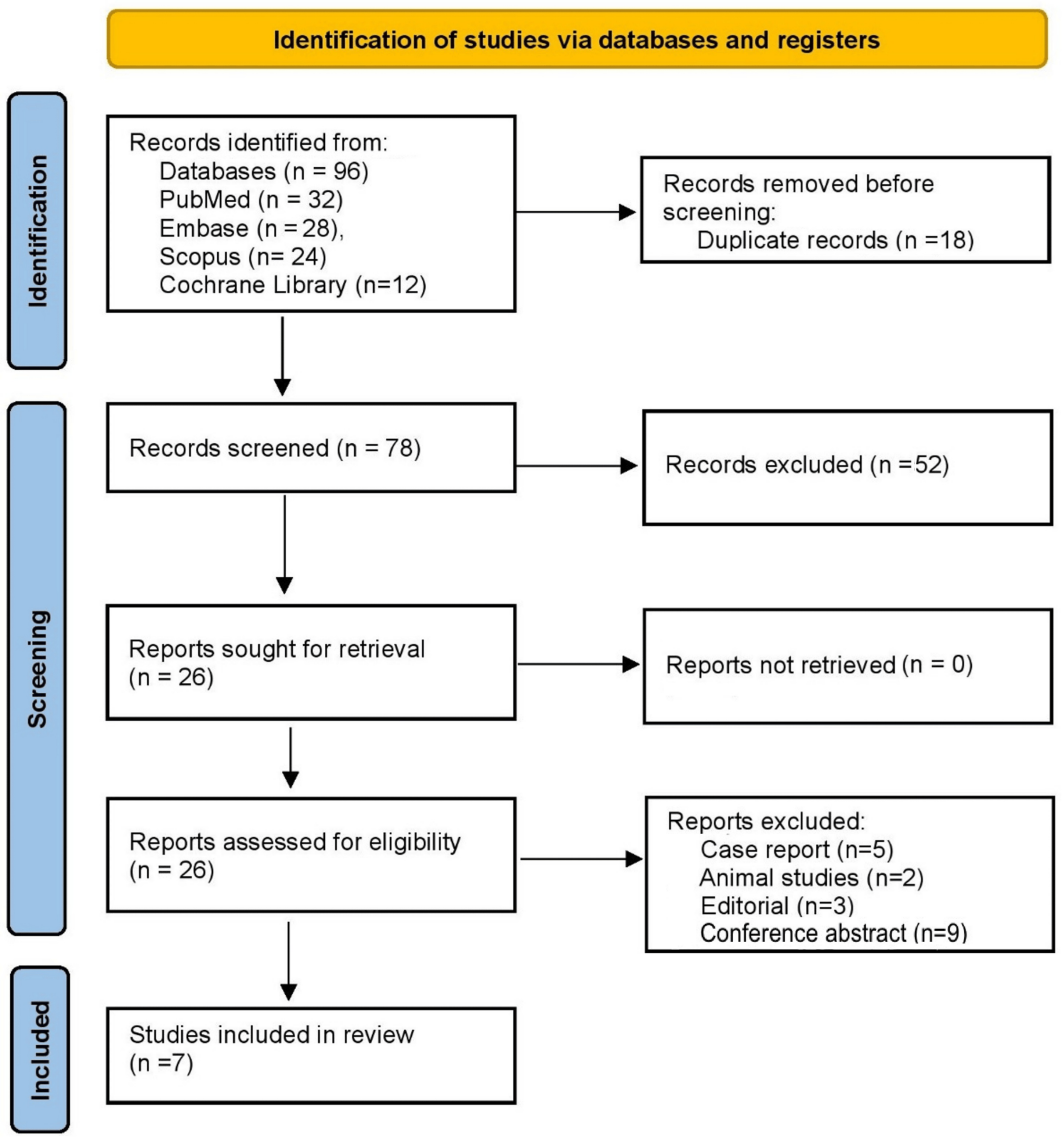
PRISMA 2020 flow diagram PRISMA: Preferred Reporting Items for Systematic Reviews and Meta-Analyses

Figure 1 shows that the database search initially retrieved 96 records (PubMed = 32, Embase = 28, Scopus = 24, Cochrane = 12). After the removal of 18 duplicates, 78 records were screened by title and abstract. Of these, 52 studies were excluded because they did not evaluate FAST in the context of hemorrhage or trauma severity classification. A total of 26 full-text articles were assessed for eligibility. Following a detailed review, 19 studies were excluded: case reports (n = 5), animal studies (n = 2), editorials and expert opinions (n = 3), and conference abstracts without sufficient outcome data (n = 9). Many of these excluded reports described FAST use for isolated diagnostic purposes but lacked prognostic evaluation or stratification outcomes relevant to hemorrhage classification. Finally, seven studies fulfilled the eligibility criteria and were included in this systematic review [14-20]. 

Characteristics of the Selected Studies

Table 1 presents the seven selected studies evaluating the diagnostic and prognostic value of FAST in trauma. Stralec et al. demonstrated that positive FAST was strongly associated with severe hemorrhage, adverse outcomes, and higher mortality [14]. Daniel et al. reported its predictive role in transfusion and operative needs [16], while Rozycki et al. confirmed high specificity (97%) for hemoperitoneum and mortality prediction [17]. Kim et al. linked positive FAST to lower systolic blood pressure (SBP), worse Glasgow Coma Scale (GCS) scores, and increased surgery or transfusion [18]. Carter et al. noted limited sensitivity but good specificity [19], and Kirkpatrick et al. showed true-positive FAST correlated with higher ISS, operative intervention, and mortality [20]. 

**Table 1:** Characteristics of the selected studies FAST: Focused Assessment with Sonography for Trauma; ISS: injury severity score; ICU: intensive care unit; SBP: systolic blood pressure; GCS: Glasgow Coma Scale; CT: computed tomography

Authors and Year	Population (P)	Exposure/Condition (I)	Comparator (C)	Outcomes (O)	Pathophysiological Findings	Prognosis	Hemorrhage Stratification	Trauma Severity
Stralec et al., 2024 [14]	527 thoracoabdominal trauma patients (prehospital)	FAST positive	FAST negative	Severe hemorrhage, ICU admission, mortality	Pleural/peritoneal free fluid	Positive FAST: higher 30-day mortality	Identified severe hemorrhage with 87% specificity	Positive FAST linked to higher ISS and interventions
Dammers et al., 2017 [15]	421 stable blunt abdominal trauma	FAST positive	FAST negative	Adverse outcome (laparotomy, embolization, death)	Free intraperitoneal fluid on CT/surgery	Positive FAST strongly correlated with adverse outcomes	Positive likelihood ratio 34.3 for hemorrhage	Higher ISS and mortality risk in positive FAST
Daniel et al., 2025 [16]	80 blunt abdominal trauma (prospective)	FAST positive	FAST negative	Surgery, ICU stay, mortality	Free fluid on ultrasound	FAST positivity predicted a worse outcome	Good stratification for massive transfusion and operative need	Correlated with trauma severity indices
Rozycki et al., 1998 [17]	1,540 trauma patients (multi-center)	FAST positive	FAST negative	Sensitivity, specificity, need for laparotomy, mortality	Hemoperitoneum detection	FAST positivity predicted emergent laparotomy and mortality	High specificity (97%) for detecting significant hemorrhage	Positive FAST aligned with higher ISS and poorer survival
Kim et al., 2022 [18]	2,758 blunt abdominal trauma	FAST positive	FAST negative	Laparotomy, mortality	Organ injuries (CT/surgery)	True-positive group: lower SBP, worse GCS	Higher laparotomy and transfusion requirements	FAST positivity associated with severe injury
Carter et al., 2015 [19]	1,671 blunt trauma patients	FAST positive	FAST negative	CT or laparotomy confirmed intra-abdominal injury	Organ lacerations, hemoperitoneum	Low sensitivity limited prognostic power	High specificity for major hemorrhage	Positive FAST associated with unstable physiology
Kirkpatrick et al., 2005 [20]	313 blunt abdominal trauma (level I trauma center)	False-negative FAST	True-positive FAST	Mortality, laparotomy, ICU stay	Free fluid/organ injury on CT/autopsy	False-negative FAST not linked to increased mortality	True-positive FAST group had higher transfusion/operative need	FAST positivity aligned with high ISS and mortality risk

Risk of Bias Assessment

Table 2 summarizes the risk of bias assessment for the included studies. Stralec et al.'s study was rated low-to-moderate risk on the NOS due to its prospective design, but with possible prehospital variability [14]. Dammers et al.'s study showed moderate risk, limited by its retrospective nature and incomplete adjustment for confounders [15]. Daniel et al.'s study had a low risk on the NOS, supported by clear prespecified outcomes, though restricted by a small sample size [16]. Rozycki et al.'s study demonstrated low risk on QUADAS-2 given its large multicenter prospective cohort, despite operator variability and older ultrasound technology [17]. Kim et al.'s study was judged moderate risk using NOS and QUADAS-2 because of its retrospective design and incomplete follow-up [18]. Carter et al.'s study carried a moderate-to-high risk on the NOS due to low sensitivity and underreporting of negative FAST [19]. Finally, Kirkpatrick et al.'s study was considered moderate risk, with detailed outcome reporting but limited external validity from its smaller cohort [20].

**Table 2:** Risk of bias assessment NOS: Newcastle-Ottawa Scale; QUADAS-2: Quality Assessment of Diagnostic Accuracy Studies, Version 2; CT: computed tomography; FAST: Focused Assessment with Sonography for Trauma

Authors and Year	Study Design	Risk of Bias Tool	Risk of Bias Rating	Justification
Stralec et al., 2024 [14]	Prospective prehospital cohort	NOS	Low to moderate	Prospective design, clear outcomes; limitation: prehospital variability, possible selection bias.
Dammers et al., 2017 [15]	Retrospective cohort	NOS	Moderate	Large sample; limitation: retrospective design, possible misclassification, incomplete adjustment for confounders.
Daniel et al., 2025 [16]	Prospective observational study	NOS	Low	Prospective, prespecified outcomes, clear definitions; limitation: single-center, small sample.
Rozycki et al., 1998 [17]	Multicenter prospective cohort	QUADAS-2	Low	Very large sample, prospective, gold-standard reference; limitation: operator variability, older ultrasound technology.
Kim et al., 2022 [18]	Retrospective multicenter cohort	NOS + QUADAS-2	Moderate	Large sample, real-world data; limitation: retrospective design, spectrum bias, incomplete follow-up.
Carter et al., 2015 [19]	Retrospective single-center cohort	NOS	Moderate to high	Consecutive patients, CT/surgery reference; limitation: very low sensitivity, retrospective design, underreporting of negative FAST.
Kirkpatrick et al., 2005 [20]	Prospective cohort	NOS	Moderate	Detailed outcome reporting, CT/autopsy confirmation; limitation: smaller cohort, external validity limited.

Discussion

This systematic review demonstrates that FAST plays a dual role as both a diagnostic and prognostic tool in trauma care. Across the seven included studies, FAST consistently showed high specificity for hemorrhage detection and provided valuable prognostic insights, linking positive findings with greater transfusion requirements, higher ISS, operative interventions, and mortality. Together, these findings highlight FAST as a cornerstone of both hemorrhage stratification and trauma severity classification. FAST’s primary diagnostic role is reflected in its high specificity for clinically significant intra-abdominal bleeding. In one of the largest multicenter studies, Rozycki et al. evaluated 1,540 trauma patients and demonstrated a specificity of 97% for hemoperitoneum, with positive scans strongly predicting laparotomy and mortality [17]. Similarly, Carter et al. confirmed FAST’s diagnostic utility in 1,671 blunt trauma patients, though its sensitivity was limited. Despite this limitation, positive findings were closely associated with unstable physiology and major hemorrhage [19]. The study by Kim et al. involving 2,758 patients further reinforced these observations, showing that true-positive FAST correlated with lower SBP, worse GCS scores, and higher rates of laparotomy and transfusion [18]. These results confirm that a positive FAST result remains a reliable diagnostic marker for significant hemorrhage in blunt trauma.

FAST is more than a diagnostic tool; it is also a prognostic indicator of adverse outcomes. Stralec et al., in a prehospital cohort of 527 thoracoabdominal trauma patients, showed that FAST positivity was associated with severe hemorrhage, higher ICU admissions, and a significant increase in 30-day mortality [14]. Dammers et al. reported similar findings in 421 hemodynamically stable blunt abdominal trauma patients, where a positive FAST carried a positive likelihood ratio of 34.3 for hemorrhage and was strongly correlated with mortality and adverse outcomes such as laparotomy and embolization [15]. These results highlight that FAST, beyond identifying bleeding, can be used to anticipate resource utilization, guide triage, and predict survival. FAST is also useful for stratifying hemorrhage severity by complementing clinical classifications such as ATLS. Daniel et al., in a prospective study of 80 blunt trauma patients, showed that FAST positivity effectively predicted worse outcomes and was strongly associated with massive transfusion requirements and surgical intervention [16]. Similarly, Kirkpatrick et al. demonstrated that true-positive FAST results correlated with higher transfusion needs, ICU admission, and mortality risk, while false-negative results were not associated with increased mortality [20]. These findings illustrate how FAST can move beyond binary interpretation and be incorporated into semi-quantitative stratification systems, such as the Kirkpatrick FAST scoring system and the Kimura and Boulanger grading system, where the number and distribution of positive zones directly correlate with blood loss and clinical severity.

FAST also integrates into trauma severity scoring systems, linking imaging findings with physiological derangement. Studies included in this review support the use of FAST in models such as the TASH score and the ABC score, which combine FAST positivity with hemodynamic variables to predict the probability of massive transfusion. For instance, Kim et al. demonstrated that patients with positive FAST results not only had more severe injuries on CT but also worse clinical status (lower SBP, worse GCS score), aligning FAST with systemic indicators of trauma severity [18]. Similarly, the findings of Dammers et al. and Stralec et al. showed that FAST can stratify stable and unstable trauma patients alike, with prognostic implications extending across populations [14,15]. Collectively, these results highlight FAST as an integral part of trauma severity classification frameworks, linking anatomical injury with outcome prediction.

The included studies reveal several important limitations. First, operator dependency remains a key challenge, as diagnostic accuracy varies with examiner skill. Second, the sensitivity of FAST is modest, particularly in detecting retroperitoneal hemorrhage or small-volume bleeding, as noted in Carter’s series [19]. Third, study heterogeneity was evident: while Rozycki et al. and Kim et al. provided large multicenter data, others, such as Daniel et al., involved smaller single-center cohorts, limiting generalizability [16-18]. Furthermore, most retrospective studies were subject to potential confounding, spectrum bias, and underreporting of negative FAST results. Lastly, while semi-quantitative scoring systems show promise, they require external validation before universal adoption.

Future research should focus on standardizing FAST scoring systems and incorporating them into widely accepted trauma severity scales. Large, multicenter prospective studies are needed to validate prognostic tools such as the Kirkpatrick and the Kimura and Boulanger grading systems. Additionally, the role of serial FAST examinations should be explored, as repeated scans may improve the detection of evolving hemorrhage and enhance prognostic accuracy. Advances in point-of-care ultrasound technology and integration of artificial intelligence for automated fluid detection and quantification may further reduce operator dependence and improve diagnostic precision. Finally, linking FAST results with comprehensive trauma registries and validated prediction scores such as TASH and ABC will refine its role in guiding transfusion, operative urgency, and trauma severity classification.

## Conclusions

FAST is a cornerstone of trauma evaluation, offering both diagnostic accuracy in identifying significant hemorrhage and prognostic value in predicting transfusion requirements, operative intervention, and mortality. Evidence from included studies demonstrates that positive FAST results correlate with unstable physiology, higher ISS, and worse clinical outcomes. When combined with hemorrhage stratification systems and trauma severity scores, FAST transcends its binary origins to become a classification and stratification tool that guides resuscitation, transfusion, and surgical decision-making. Despite limitations in sensitivity and operator variability, ongoing research, technological advances, and standardized scoring systems will further strengthen the role of FAST in hemorrhage stratification and trauma severity classification.
